# Vasopressors and inotropes in cardiogenic shock: is there room for “adrenaline resuscitation”?

**DOI:** 10.1186/s13054-016-1459-2

**Published:** 2016-09-27

**Authors:** Nuccia Morici, Miriam Stucchi, Alice Sacco, Maurizio A. Bottiroli, Fabrizio Oliva, on behalf of the AltShock group, Stefano Carugo, Diego Castini, Emanuele Catena, Manlio Cipriani, Elena Corrada, Maria Frigerio, Maria Pia Gagliardone, Andrea Garascia, Francesco Gentile, Antonio Mafrici, Filippo Milazzo, Marco Negrini, Federico Pappalardo, Claudio Russo, Michele Senni, Romano Giuseppe Seregni

**Affiliations:** 1 De Gasperis Cardio Center, Niguarda Ca’ Granda Hospital, Milan, Italy; 2 Anestesia e Rianimazione 3, Niguarda Ca’ Granda Hospital, Milan, Italy

We read with interest the paper of Tarvasmäki et al. [1] regarding the role of inotropes and vasopressors in patients with cardiogenic shock. The authors should be congratulated for their effort of prospectively collecting a huge amount of data in one of the most challenging settings. However, too much emphasis seems to be placed on the authors’ conclusions, especially taking into account some of the study’s limitations.

Right from the start of the paper, a fearsome association between adrenaline and short-term mortality in the generic setting of cardiogenic shock is described. However, up to 80 % of the patients enrolled in the study had an acute coronary syndrome as the cause leading to shock and this should have been emphasized [2].

Adrenaline doses (maximum infusion rate 0.22 mcg/kg/min (interquartile range 0.10–0.36)) were abnormally high. It is well known that, depending on vascular beds and concentration, adrenaline may induce either vascular dilatation or contraction, with the vasopressor effects arising at higher doses [3]. These effects might be extremely amplified when coupled with other vasopressors, leading to impaired organ functions. In this study, patients receiving adrenaline were also more likely to receive higher doses of noradrenaline and dopamine.

Furthermore, patients receiving adrenaline were more frequently resuscitated prior to admission, had a higher incidence of low output state, and worse renal function at admission. A more frequent recourse to a mechanical assist device was also present in these patients. Considering the small sample size (40 patients received adrenaline), might propensity score and multivariable analysis take account of all these confounding and effect modifiers? It is well known that researchers should use extreme caution when interpreting the results of analyses performed including a propensity score as a covariate in a multivariable model [4].

Looking at the unadjusted odds ratios reported in their Fig. 1 [1], we were impressed by the confidence intervals. Levosimendan was used in 52 patients: this sample is small but the effect estimation is accurate because these patients were, presumably, clinically selected. Noradrenaline was used in 162 patients: this sample is larger but the effect estimation is less accurate because, supposedly, these patients were a more heterogeneous group. What about the patients receiving adrenaline? Were they at the extreme spectrum of the population enrolled?

We believe there is not enough evidence to promote a link between adrenaline administration and increased death rates. Conversely, low to mid doses of vasopressors might be considered part of an integrated approach but only when massive myocardial damage has not yet occurred.

## Abbreviation

CS: Cardiogenic shock
