# Supplementary material for: Vasopressors and inotropes in cardiogenic shock: is there room for “adrenaline resuscitation”?
Source: Crit Care. 2016 Sep 27;20:302. doi: 10.1186/s13054-016-1459-2 (PMC5039816; doi:10.1186/s13054-016-1459-2)
Supplement: Related texts

# Reply: Authors’ response to “Vasopressors and inotropes in cardiogenic shock: is there room for ‘adrenaline resuscitation’?”

**Authors:** Tuukka Tarvasmäki, Johan Lassus, Alexandre Mebazaa, Veli-Pekka Harjola

We thank Morici and colleagues for their interest in our paper and acknowledging our work. They point out that most patients in our study had cardiogenic shock (CS) caused by acute coronary syndrome. This is clearly stated in the results and also described in the previously published paper on the characteristics and outcome of this prospectively enrolled cohort of CS [5].

The dose of adrenaline and concomitant use of other vasopressors and inotropes may indeed be subject to variation according to local experience and practices. In a contemporary randomized controlled trial in CS, IABP-SHOCK II, the median dose of adrenaline was 0.3 μg/kg/min (Table S1 in the Supplementary Appendix of [6]), which is very similar to the dose recorded in the CardShock study. However, the association of adrenaline with mortality was not dependent on the doses of other vasopressors. The characteristics of patients treated with and without adrenaline are also shown in Table 1 in our paper [1] and mortality analyses were adjusted for differences. Mechanical assist devices other than an intra-aortic balloon pump were used in very few patients overall.

We are aware of the limitations regarding propensity score adjustment. Therefore, we performed additional propensity score matching as a sensitivity analysis, which confirmed the finding. In addition, further adjustment with the use of intra-aortic balloon pump or other mechanical assist devices did not change the results. While the study population is of limited size and the estimates of treatment effects may be susceptible to bias by unknown or unmeasured variables, we think that the results are consistent. All in all, while our study does not prove causality, it does raise safety concerns about using adrenaline in CS.
